# Study on Fire Behavior, Thermal Stability and Degradation Kinetics of Thiol-Ene with Poly(aminopropyl/phenyl)silsesquioxane

**DOI:** 10.3390/polym14061142

**Published:** 2022-03-12

**Authors:** Jiangbo Wang

**Affiliations:** School of Materials and Chemical Engineering, Ningbo University of Technology, Ningbo 315211, China; jiangbowang@nbut.edu.cn; Tel.: +86-0574-87081240

**Keywords:** flame retardancy, poly(aminopropyl/phenyl)silsesquioxane, thiol-ene, kinetics, activation energy

## Abstract

In this article, the flame retardant poly(aminopropyl/phenyl)silsesquioxane (PA) was incorporated into thiol-ene (TE), to obtain a flame-retardant thiol-ene (FRTE) composite. The cone calorimeter (CONE) measurement results showed that, compared with neat TE, the peak of heat release rate (PHRR) and total heat release (THR) of FRTE have decreased by almost 23.7% and 14.5%, respectively. Thermogravimetric analysis (TGA) results further confirmed that the flame retardant PA could induce the initial thermal degradation of TE, and increased the amounts of residual char. Moreover, the activation energies of FRTE were calculated through the Kissinger and Flynn–Wall–Ozawa methods. Compared with the neat TE, the activation energies of FRTE were raised by the addition of PA. It indicated that the flame retardant PA promoted cross-linking reactions of TE, to form a compact char layer and retarded further the thermal degradation of the polymer matrix.

## 1. Introduction

UV-photopolymerization is a simple and efficient way of generating cross-linked networks. Due to the advantages of solvent-free, environment-friendly, all active ingredients, and rapid curing under UV irradiation, UV-curing film has strong potential application in the field of coatings. A wide variety of monomers (including multifunctional acrylate and methacrylate monomers) have been found to undergo rapid photopolymerization under UV light, with the right amount of photoinitiator [1,2,3]. However, there are still many problems in the above monomer systems, such as the fact that it is unstable in oxygen, has uneven crosslinking, large internal stress in polymerization, is easy to cause volume shrinkage, and so on [4,5,6,7,8].

Thiol-ene (TE) photopolymerization is a novel photopolymerization, based on click chemistry, which is different from the step-growth reaction mechanism of an acrylate-based photopolymerization system. It has the characteristics of uniform cross-linking network, gel point delay, low volume shrinkage and low stress, which overcome the defects of previous conventional photopolymerization systems. In addition, the rate of the thiol-ene addition reaction is very fast, which is almost equal to the photopolymerization of acrylate under inert conditions. Conventional radical addition polymerization is difficult to carry out in the presence of oxygen, but the thiol-ene reaction is different from this. It can occur in the presence of oxygen and will not be affected. [9,10,11,12,13]. However, the thiol-ene polymer, like most organic polymers, is deficient in flame retardancy. Therefore, adding some additional substances into thiol-ene is necessary to reduce its flammability [14,15].

Halogen flame retardance, the earliest used flame retardant, is an important kind of organic flame retardant at present. With low price and additional excellent stability and compatibility, it has become one of the most used flame retardants in the world [16,17]. However, the halogen gas released from the combustion of halogen containing polymers will generate corrosive, harmful gas (hydrogen halide), when combined with water vapor, causing corrosion to some equipment and buildings. Halogen flame retardants will release strong carcinogens, such as dioxin and benzofuran, after combustion, affecting normal human metabolism and seriously damaging the environment [18,19,20].

The use of halogen-free flame retardants has become the development trend of polymer flame retardants. As a high-efficiency, smokeless, low-toxicity and pollution-free flame retardant, the phosphate flame retardant has attracted the interest of many researchers. At present, remarkable achievements have been made in synthesis and application. However, most phosphate flame retardants also have some disadvantages, such as high volatility, poor heat resistance, poor compatibility, and dripping during combustion. Inorganic flame retardants mainly include hydroxide (aluminum hydroxide, magnesium hydroxide), red phosphorus, tin series and borate (zinc borate) [21,22,23,24]. Inorganic flame retardants not only have a flame retardant effect, but also have a smoke suppression effect, and can inhibit the formation of hydrogen chloride. Inorganic flame retardants are widely used because they are non-toxic and non-corrosive. Today, with the increasing requirements of environmental protection, inorganic flame retardants show strong competitiveness and development potential. The disadvantage is that inorganic flame retardants generally have relatively large addition and low flame-retardant efficiency, which seriously damage other properties of the polymer matrix. As reported in the literature, polysiloxane has been demonstrated as an effective and ‘environment friendly’ flame retardant, for various polymers. Silicon, due to its low surface energy, migrates easily to the surface of the polymer matrix during combustion. Thus, the thermal degradation of the polymer can be effectively prevented, by forming a protective layer with excellent heat resistance [25,26,27,28]. However, as far as we know, no one has studied the effect of polysiloxane to enhance the fire behavior and thermal property of the thiol-ene system.

Thus, in this paper, poly(aminopropyl/phenyl)silsesquioxane (PA) was incorporated into thiol-ene to enhance the flame retardancy of the composites. We chose PA because the phenyl groups, in their structure, have excellent char-forming properties. Additionally, the amino group forms nitrogen during combustion, which also has a flame-retardant effect. Then, the fire behavior and thermal degradation behavior of siliconized-modified thiol-ene were investigated by cone calorimeter measurement and thermogravimetric analysis (TGA), respectively.

## 2. Materials and Methods

### 2.1. Materials

Trimethylolpropane tris(3-mercaptopropionate) (3T) was supplied by Bruno Bock Chemische Fabrik GmbH & Co. (Marschacht, Germany). Tetramethylammonium hydroxide (TMAOH) and phenyltriethoxysilane (PTES) were supplied by Alfa Aesar Chemical Reagent Co. Ltd. (Tewksbury, MA, USA). Sigma-Aldrich Reagent Co. Ltd. (St. Louis, MO, USA) supplied 2,2-Dimethoxy-2-phenylacetophenone (DMPA), pentaerythritol allyl ether (TAE), (3-aminopropyl)triethoxysilane (APS) and ethyl alcohol (EtOH) were all used as received.

### 2.2. Synthesis of Poly(aminopropyl/phenyl)silsesquioxane (PA)

**Figure 1:**
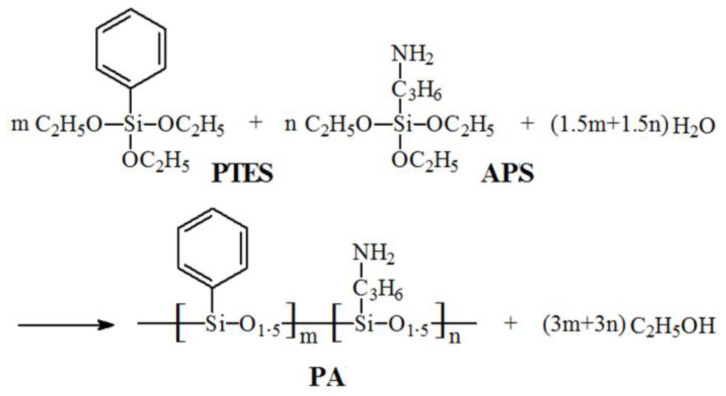
Synthesis of PA.

As shown in Figure 1, the synthesis of PA was based on previous publications and the specific method was as follows [29,30]: EtOH (75 mL), distilled water (25 mL) and TMAOH (1 mL) were added into a 250 mL flask. Then, PTES and APS at different molar ratios were mixed in the above solution, accounting for 10 wt% of the total. Stirring was stopped after 8 h and left overnight. The supernatant was removed and the precipitate condensate was collected. It was then pumped and filtered with EtOH/distilled water (3/1 by volume) and washed with anhydrous EtOH. The product was dried in vacuum for 20 h at room temperature to obtain PA.

### 2.3. Preparation of TE Composites

For the composite preparation, 1 wt% DMPA was first dissolved in 3T and ultrasound was performed for 30 min. Then, equal amounts of TAE with 3T and PA (5 wt% of the total amount) were added to the mixture and stirred evenly. The mixture was further mixed with an ultrasonic device and the bubbles were removed (30 min). TE/PA (FRTE) composites were prepared by UV curing after pouring the mixture onto the glass substrate. For comparison, TE was prepared under the same technological conditions.

### 2.4. Characterization and Measurement

Cone calorimeter measurement was carried out using an FTT Conical Calorimeter (Fire Testing Technology Ltd., East Grinstead, West Sussex, UK) according to ASTM E1354. The heat flux was 50 kW/m^2^ and the specimen size was 100 × 100 × 3 mm^3^. All specimens were measured in three groups and then averaged. Thermogravimetric analysis (TGA) was performed on the Q5000 TA Thermogravimetric Analyzer (TA Instrument Corp., New Castle, DE, USA). In a nitrogen atmosphere, about 10 mg of the sample was heated from 50 °C to 600 °C at 10 °C/min heating rate.

### 2.5. Thermal Degradation Theory

When studying the thermal transformation kinetics of solid chemical reactions, it is generally based on the following reaction rate [31,32]:

(1) $$r=\frac{da}{dt}=kf(a)$$

where, *r* is the degradation rate, *a* is the conversion degree, *t* is the time, *k* is the rate constant, *f(a)* is the reaction model. It is generally assumed that *k* obeys the Arrhenius equation:

(2) k=Aexp(−E/RT)

where, *A* is the pre-exponential factor, *E* is the activation energy, *R* is the universal gas constant and *T* is the temperature.

The influence relationship between degradation rate and temperature and sample weight change can be expressed as:

(3) $$\frac{da}{dt}=Af(a)\exp(-E/RT)$$

Equation (3) can also be used in its integral form. Under isothermal conditions, the integral form is:

(4) lnt=E/RT−ln[A/g(x)]

For non-isothermal degradation, Equation (3) becomes:

(5) $$\frac{da}{dT}=(A/\beta )f(a)\exp(-E/RT)$$

where, β is the heating rate ($\beta =\frac{dT}{dt}$), g(x) is the mechanism integrated forms ($g(x)=\int_{0}^{a}\frac{da}{f(a)}$).

(1) Kissinger method [33]

The equation of the Kissinger method can be expressed as follows:

(6) $$\ln(\frac{\beta }{T_{\max}^{2}})=\ln(\frac{AR}{E})-\frac{E}{RT_{\max}}$$

where, $T_{\max}$ is the temperature of the peak rate.

The temperature of peak rate is determined by the DTG curves at different heating rates. Then draw with 1/$T_{\max}$ as the abscissa and $\ln(\beta /T_{\max}^{2})$ as the ordinate and fitting a straight line. The activation energy can be calculated from the slope of the line by the Kissinger equation.

(2) Flynn–Wall–Ozawa method [34,35]

The equation of the Flynn–Wall–Ozawa method is as follows:

(7) $$\mathrm{lg}(\beta )=\mathrm{lg}AE/g(a)R-2.315-0.457\frac{E}{RT}$$

As can be seen from the above equation, variable lg(β) is linearly proportional to variable 1/T. The activation energy for any particular degree of degradation can be obtained by calculating the slope of the lg(β) − 1/T plots.

## 3. Results and Discussion

### 3.1. Flame Retardancy

**Figure 2:**
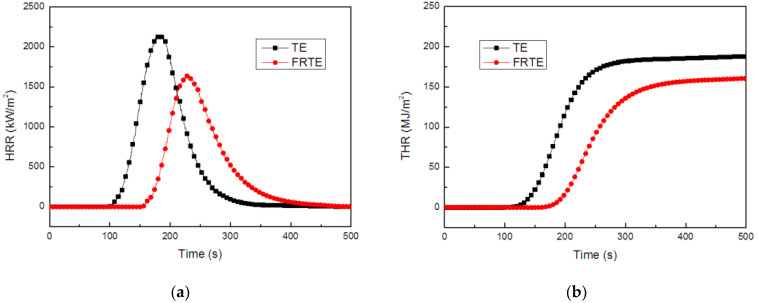
HRR (**a**) and THR (**b**) curves of TE composites.

There are many traditional fire hazard testing methods, but most of them use small instruments to test the performance, which is far from the actual situation when a fire occurs. The cone calorimeter is mainly based on the principle of oxygen consumption for testing. It provides a way to measure multiple different parameters in the same experiment. It has been shown that the cone calorimeter test results have a very good correlation with the parameters obtained from large-scale fire tests. Thus, it can be used to predict the burning behavior of materials in real fires [36]. The cone calorimeter of TE composites is presented in Figure 2. It could be obtained that the peak of heat release rate (PHRR) for the neat TE reached 2152.4 kW/m^2^, which presented a very sharp heat release rate (HRR) curve and the combustion was complete after 321 s. Compared with neat TE, the incorporation of 5 wt% PA led to a strong reduction in PHRR, which reached a value of 1642.8 kW/m^2^ and the PHRR was reduced by nearly 23.7%. The reduction in HRR was accompanied by a prolongation of burning time (from 321 to 409 s). Moreover, it was clear that the total heat release (THR) evidently decreased (from 188.0 to 160.7 MJ/m^2^) for the FRTE composite, compared with the neat TE matrix.

The Flame Retardancy Index (FRI) was always used to evaluate the flame retardancy of resin systems [37,38]. The calculation equation of FRI is as follows:

(8) $$\mathrm{FRI}=\frac{{\left[\mathrm{THR}\times \left(\frac{\mathrm{PHRR}}{\mathrm{TTI}}\right)\right]}_{\mathrm{TE}}}{{\left[\mathrm{THR}\times \left(\frac{\mathrm{PHRR}}{\mathrm{TTI}}\right)\right]}_{\mathrm{FRTE}}}$$

As shown in Table 1, the FRI value of PA was 2.31 after calculating the relevant parameters, which exhibited “good” flame retardancy performance.

**Figure 3:**
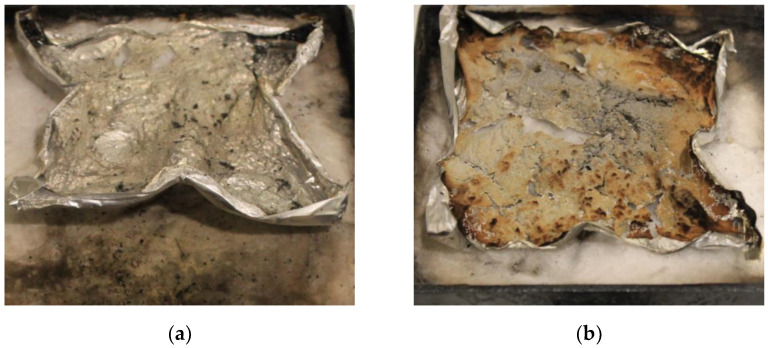
Residual char images of TE (**a**) and FRTE (**b**) after CONE measurement.

The morphology of the char residue in the cone calorimeter after combustion is presented in Figure 3. It can be seen that the neat TE was fully burned and there was practically no residue (Figure 3a). In contrast, the amount of FRTE residue was large and the color was light yellow (Figure 3b). It was particularly important that the residue formed a compact and continual char layer. This obviously helps prevent the passage of heat and combustible substances in the fire and, finally, the flame retardancy of the polymer was raised.

### 3.2. Thermal Stability

**Figure 4:**
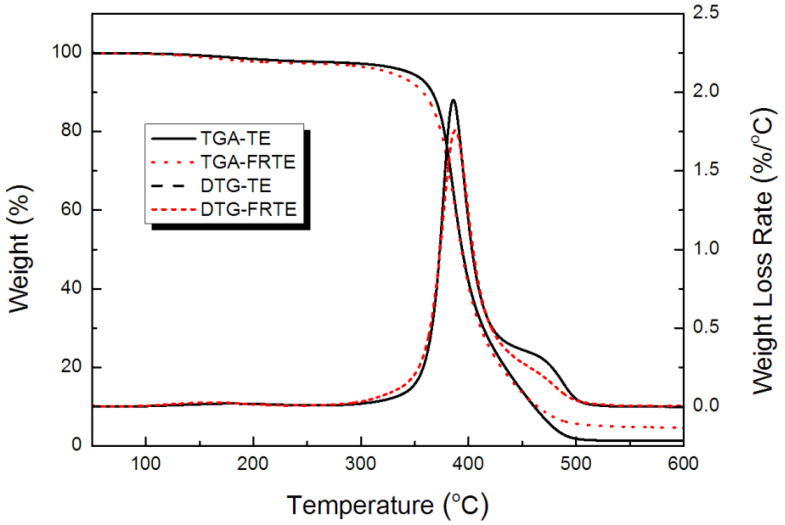
Thermal stability of TE composites.

The TGA/DTG curves for the degradation of the TE composites, at a heating ramp rate of 10 °C/min in nitrogen, are presented in Figure 4, and the data are listed in Table 2. It revealed that the onset degradation temperature (*T_5wt%_*) of FRTE was much lower than that of neat TE. The *T_5wt%_* for TE was 345.7 °C, but 327.3 °C for the FRTE, indicating that the onset degradation temperature of TE tended to decrease with the incorporation of PA. The major degradation in the FRTE occurred between 300–500 °C, which was similar to that of TE.

The char residues of the FRTE at 600 °C were higher than that of TE, and the amount of solid residue shifted from 1.33 wt% (for TE) to 4.60 wt% (for FRTE) of the initial weight. The phenyl groups in the structure had excellent char-forming properties and aminopropyl groups could promote the crosslinking reactions during the thermal degradation process. In addition to that, nitrogen could be formed from the amino group during combustion, which also has a flame-retardant effect. This result further confirmed that the branched silicone with aminopropyl and phenyl could induce the formation of the char layer, which might play an important role for the flame retardancy of the FRTE composite [39,40].

### 3.3. Thermal Degradation Kinetics

**Figure 5:**
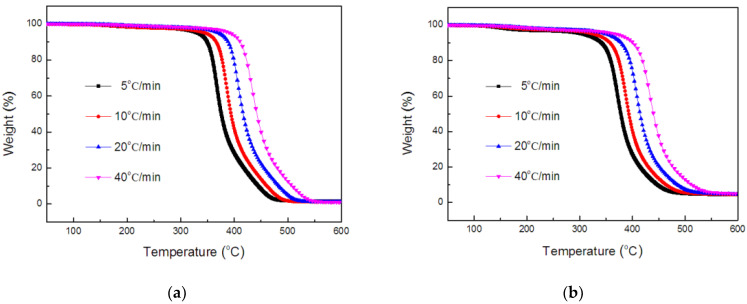
TGA curves of TE (**a**) and FRTE (**b**) composites.

**Figure 6:**
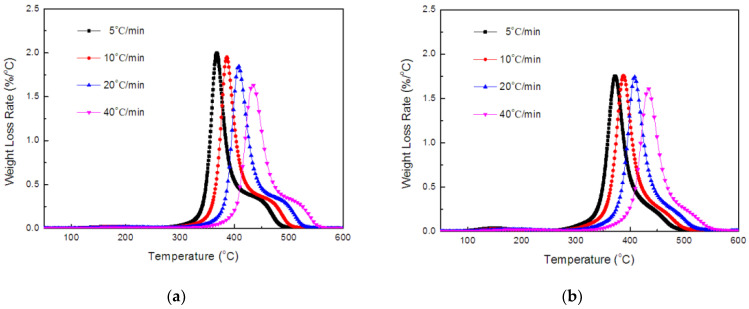
DTG curves of TE (**a**) and FRTE (**b**) composites.

The TGA and DTG curves of the TE composites, at the heating rates of 5, 10, 20 and 40 °C/min, are shown in Figure 5 and Figure 6. The curves revealed the different profiles, depending on heating rate, and two weight-loss stages occurred during degradation, which was consistent with the literature report [41]. The first major degradation in the TE composites emerged in a temperature range of 300–450 °C, whereas the second stage degradation was observed above 450 °C. The temperature of the peak rate (*T_max_*) of the TE composites increased progressively as the heating rate increased. Generally, with the increase in heating rate, the time required for the sample to reach a certain temperature is shortened. Therefore, it could be seen from Figure 5 that, when the heating rate gradually increased from 5 °C/min to 40 °C/min, the TGA curve of the sample also moved to a higher temperature.

**Figure 7:**
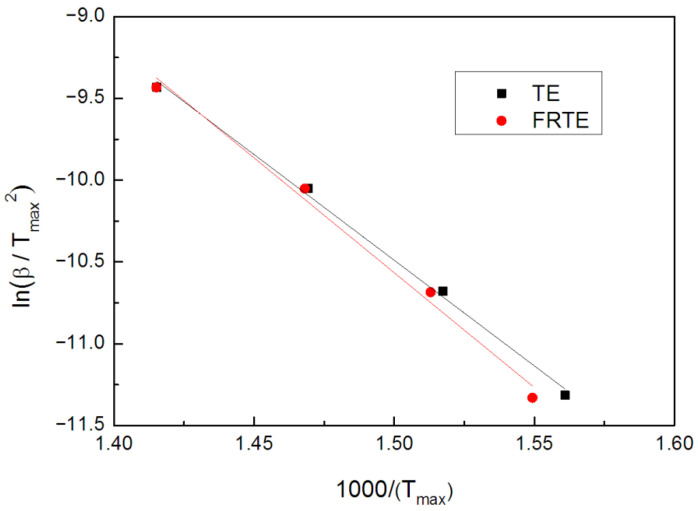
The curves of $\ln(\frac{\beta }{T_{\max}^{2}})$ vs. $\frac{1}{T_{\max}}$ of TE and FRTE.

Figure 7 presents the Kissinger plots of $\ln(\beta /T_{\max}^{2})$ versus 1000/$T_{\max}$ for TE composites. The kinetic parameters of the first stage in thermal degradation, calculated by the Kissinger method, are summarized and compared in Table 3.

The kinetic parameters of TE changed with the incorporation of PA. For FRTE, the values of activation energy and *lnA* were 116.7 kJ/mol and 13.1/min, respectively, which were significantly higher than those of neat TE. In general, the incorporation of flame-retardant PA enhanced the thermal stability of TE [42].

The Flynn–Wall–Ozawa method is another kinetics analysis method and was used in this study. Compared with the Kissinger method, the Flynn–Wall–Ozawa method can analyze the change in activation energy of a flame retardant system in the whole thermal degradation process, through simple TGA data processing, and can, therefore, obtain more comprehensive and complete kinetic data.

**Figure 8:**
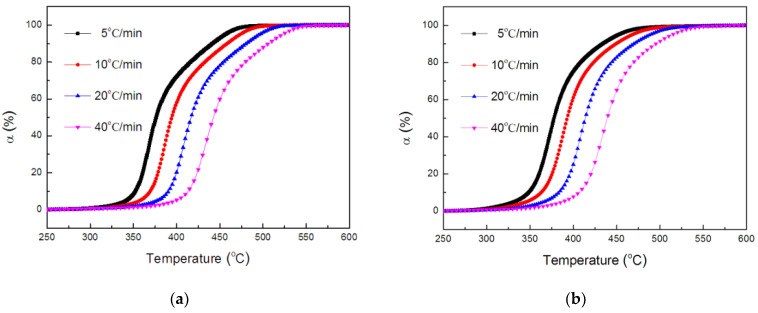
Conversion of TE (**a**) and FRTE (**b**) as a function of temperature.

Based on the data in Figure 4, and the equation of $a=\frac{w_{0}-w_{t}}{w_{0}-w_{\infty }}$ (*w*_0_ is the initial weight of the sample, *w_t_* is the sample weight at any temperature *t*, *w_∞_* is the final sample weight), the conversion degree as a function of temperature, relative to the decomposition of the TE and FRTE systems, can be calculated, as exhibited in Figure 8.

The activation energies of the thermal degradation for the TE composites could be calculated through Equation (7). The conversion values were 0.02, 0.05, 0.10, 0.20, 0.30, 0.40, 0.50, 0.60, 0.70, 0.80, 0.90, 0.95 and 0.98.

**Figure 9:**
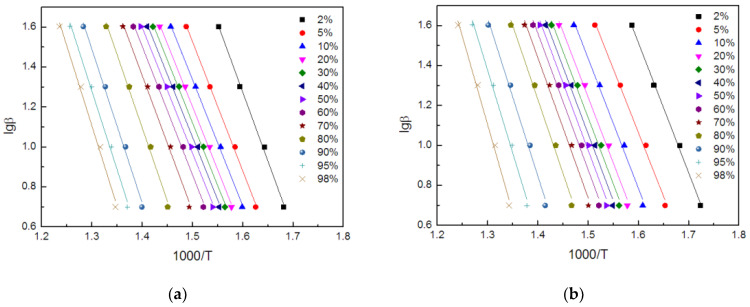
The curves of lg(β) vs. 1000/*T* of TE (**a**) and FRTE (**b**).

**Figure 10:**
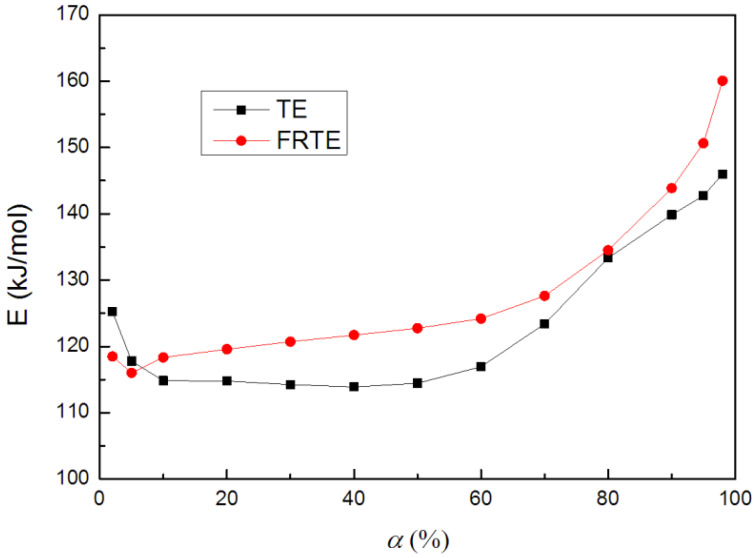
Activation energy curves by Flynn–Wall–Ozawa method.

For the fitting straight lines, obtained in Figure 9, their R2 values were both higher than 99%. This means that the Flynn–Wall–Ozawa method was suitable for this research system. Moreover, the fitting lines, corresponding to TE and FRTE, were relatively parallel, which indicated that the research system should correspond to a single reaction mechanism. The activation energy curves are presented in Figure 10.

As seen, the same tendency as for the results from the Flynn–Wall–Ozawa method was obtained. A decrease in the activation energy, with the increasing conversion in the initial degradation stage (2~10%), was found. The activation energy of FRTE at 5% conversion was around 116.0 kJ/mol, whereas that of neat TE was 117.8 kJ/mol. It was reported that the earlier thermal degradation of polymer always occurred, due to the degradation of polysiloxane at a lower temperature [43]. Then, the activation energies of neat TE and FRTE both increased with increasing conversion. With the increase in activation energy, the thermal stability of the polymer was improved, and the degradation became difficult, which indicated that the flame retardancy of the polymer was improved. However, for FRTE, the incorporation of PA led to activation energy greater than that of neat TE (α ≥ 10%). From these values, mean values of 124.4 kJ/mol and 129.1 kJ/mol were found for neat TE and FRTE, respectively, which was in best agreement with those obtained using the Kissinger method [44]. The above results indicated that the Si-C_3_H_6_NH_2_ bond and Si-Ph bond of flame retardant PA may form some silyl radicals or siloxane derivatives, which could react with TE or the evolved products of TE. Thus, the cross-linking reactions were promoted to form a compact char layer in the FRTE composite and further pyrolysis during the thermal degradation process was retarded.

## 4. Conclusions

A flame-retardant composite FRTE has been successfully prepared by the incorporation of poly(aminopropyl/phenyl)silsesquioxane into a thiol-ene matrix. The results of the cone calorimeter and TGA measurements showed that, compared with neat TE, the flame retardancy and thermal stability of FRTE were improved. Specifically, the PHRR and THR of FRTE were reduced by almost 23.7% and 14.5%, and the amounts of residual char were increased. Furthermore, the results from both the Kissinger and Flynn–Wall–Ozawa methods showed that the activation energies of FRTE were enhanced by the incorporation of PA, which indicated that the branched silicone with aminopropyl and phenyl promoted cross-linking reactions of TE, to form a compact char layer, and retarded further pyrolysis during the thermal degradation process of the polymer matrix.

## Figures and Tables

**Table 1:** The parameters obtained from the cone calorimeter measurement.

Sample	PHRR (kW/m^2^)	THR (MJ/m^2^)	TTI (s)	FRI	Flame Retardancy Performance
TE	2152.4	188.0	102.5	-	-
FRTE	1642.8	160.7	154.5	2.31	good

**Table 2:** TGA data of TE composites.

Sample	Temperature (°C)		Peak Rate (wt%/°C)	Residue Char (wt%)
	*T_5wt%_*	*T_max_*		
TE	345.7	385.9	1.95	1.33
FRTE	327.3	387.8	1.76	4.60

**Table 3:** Kinetic data using the Kissinger method.

Sample	Temperature (°C)				*E* (kJ/mol)	*lnA* (1/min)
	5 °C/min	10 °C/min	20 °C/min	40 °C/min		
TE	367.5	385.9	407.5	433.4	107.4	11.4
FRTE	372.3	387.8	408.0	433.5	116.7	13.1

## Data Availability

The data used to support the findings of this study are available from the corresponding author upon request.
